# Case Report: Myomatous erythrocytosis syndrome presenting as rapid growth of an extra-uterine mass

**DOI:** 10.3389/fsurg.2022.950358

**Published:** 2022-08-02

**Authors:** Shao-Jing Wang, Yun-An Chen, Yu-Hsiang Shih, Ming-Jer Chen, Chien-Hsing Lu

**Affiliations:** ^1^ Department of Obstetrics and Gynecology, Taichung Veterans General Hospital, Taichung, Taiwan; ^2^ Department of Pathology & Laboratory Medicine, Taichung Veterans General Hospital, Taichung, Taiwan; ^3^ Institute of Biomedical Sciences, National Chung Hsing University, Taichung, Taiwan; ^4^ Rong-Hsing Research Center for Translational Medicine, National Chung Hsing University, Taichung, Taiwan; ^5^ Department of Obstetrics and Gynecology, National Yang-Ming University School of Medicine, Taipei, Taiwan

**Keywords:** ectopic erythropoiesis, myomatous erythrocytosis syndrome, uterine fibroids, broad ligament, leiomyoma

## Abstract

**Objective:**

To report a case of myomatous erythrocytosis syndrome (MES) with an extra-uterine manifestation.

**Case report:**

A 43-year-old woman presented with progressive abdominal distension and rapid enlargement of a pelvic mass. Upon survey, a high-level of hemoglobin (19.0 g/dl) was documented. The initial impression was an ovarian malignancy, but uterine sarcoma could not be ruled out because of its rapid growth. However, during exploratory laparotomy, the pelvic mass was found to be a 31 cm broad ligament leiomyoma; which is extremely rare for its size and location. The specimen was further studied immunohistochemically, which revealed excessive expressions of erythropoietin and erythropoietin receptors in addition to the diffusely matured blood vessels in the myoma tissue. The patient’s hemoglobin level resumed to normal three months post-surgery. The diagnosis of MES was confirmed both clinically and histologically.

**Conclusion:**

A correct preoperative diagnosis is challenging when MES manifests as an extrauterine mass. The coexistence of MES should be considered in the management of all leiomyoma with polycythemia, regardless of locations.

## Introduction

Erythropoietin (Epo) is produced by interstitial fibroblasts in the kidney. Polycythemia, defined as an increased hemoglobin level (>16 g/dl) or increased hematocrit (>48%) in women, can result from tumor-induced ectopic Epo production. Myomatous erythrocytosis syndrome (MES), characterized by erythrocytosis associated with uterine leiomyoma, is a rare condition with less than 60 cases reported till date (1). Excessive erythropoiesis is associated with an increased risk of thromboembolic events, and prophylactic measurements may be required (2). Here, we report a case of MES with an extra-uterine manifestation.

## Case report

**Figure 1:**
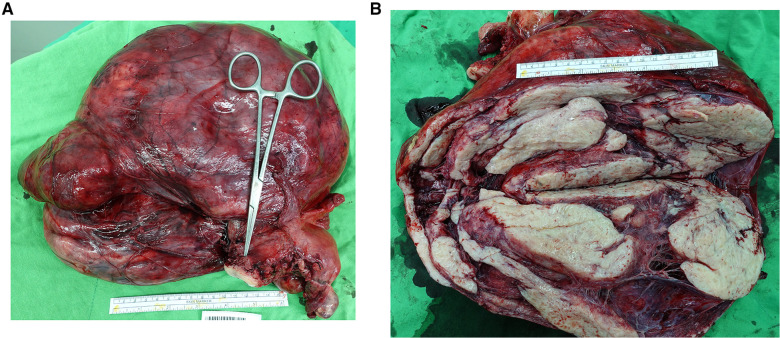
(**A,B**) The gross picture of the broad ligament myoma and incised specimen. The mass was found to have originated from the right broad ligament. The cervix was pointed by the tip of the Kelly clamp (**A**). The leiomyoma was throughout solid and presented with only a small portion of degeneration (**B**).

A 43 year old Taiwanese woman, gravida 2, para 2, reported no remarkable surgical and medical history prior to this event. She had been experiencing progressive abdominal distension and a body weight loss from 57 to 52 kg over the last six months. Upon physical examination, the abdominal mass appeared enlarged similar to seven gestational months. Computerized tomography scan revealed a 30 cm heterogeneous mass occupying the entire pelvic space, which was too large for anatomic distinction. The initial laboratory survey revealed a normal CA-125 level, a slightly elevated level of lactate dehydrogenase at 292 U/L (reference range: 120–240 U/L), and an elevated hemoglobin level of 19.0 g/dl. The first impression was an ovarian tumor, but a uterine sarcoma was not ruled out owing to its rapid growth. Exploratory laparotomy was subsequently arranged. During surgery, the pelvic mass was found to have originated from the right broad ligament, while the right ovary had a 3 cm dermoid cyst (Figures 1A,B). Total hysterectomy and bilateral salpingo-oophorectomy were performed with an estimated blood loss of 3,740 ml.

**Figure 2:**
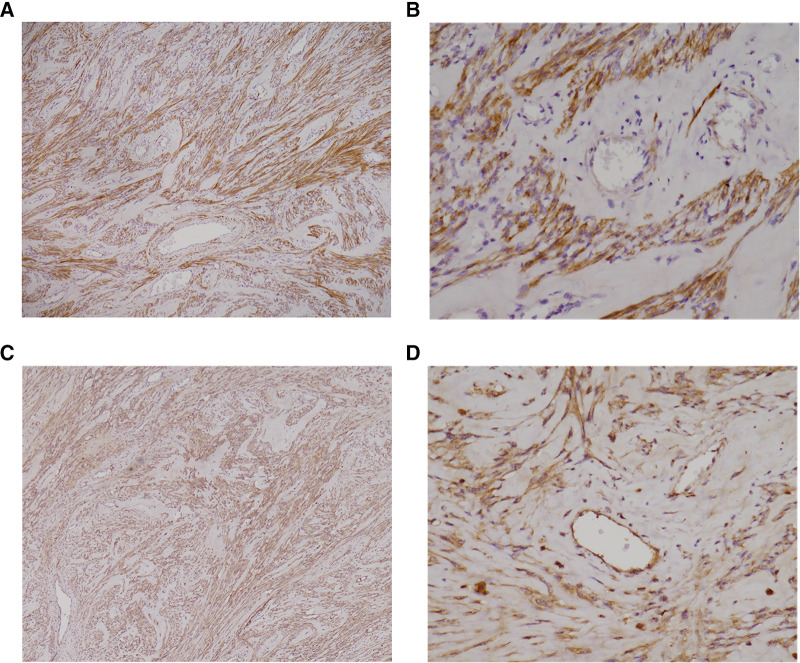
(**A–D**): Immunohistochemical staining revealed excessive Epo and Epo-R expression of the broad ligament myoma. Immunochemical staining showed diffuse (>50%) Epo expression with moderate to strong staining intensity over the myomatous cells (**A**, 40×), whereas the endothelial cells were negatively stained (**B**, 200×). A diffuse (>50%) Epo-R expression with moderate staining intensity was found over the myomatous cells (**C**, 40×). The endothelial cells also presented with diffuse, moderate to weak staining intensity (**D**, 200×). EPO, erythropoietin; Epo-R, erythropoietin receptor.

**Figure 3:**
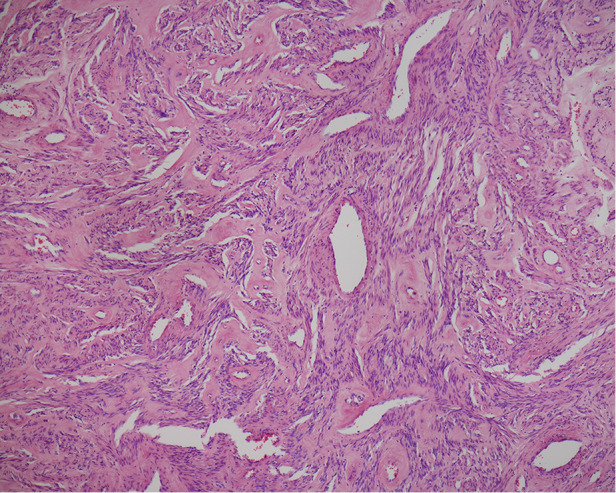
H&E staining showing wide distribution of mature vessels within the myomatous tissue. Under H&E stain (40×), the myomatous tissue presented with a vast distribution of mature vessels. The vessels bear the characteristics of dilated veins and arteries with smooth muscle layers and a luminal space of >50 µm in minimum diameter. H&E, hematoxylin and eosin.

The resected specimen had a size of 31 × 25 × 12.5 cm^3^; and under histological examination, a leiomyoma originating from the broad ligament was confirmed. To clarify the association between the myomatous tissue and erythrocytosis, immunohistochemical (IHC) staining was performed with antibodies against erythropoietin (rabbit polyclonal antibody, AF5190, Affinity Biosciences) and erythropoietin receptor (Epo-R) (rabbit polyclonal antibody, A2917, ABclonal Science). A diffuse Epo expression with moderate to strong staining intensity appeared over the myomatous tissue, but the staining was negative for the endothelial cells (Figures 2A,B). Both the myomatous and endothelial cells were positive for Epo-R immunostaining (Figures 2C,D). We also noticed an extensive distribution of mature blood vessels in the specimen under hematoxylin and eosin (H&E) stain (Figure 3).

The patient recovered well and was discharged within five days after surgery. Three months post-surgery, her hemoglobin level returned to normal (13.5 g/dl), and continued to be within the normal range. Therefore, the diagnosis of MES associated with a broad ligament leiomyoma was confirmed both clinically and histologically.

## Discussion

In 1957, Fleming et al. (3) proposed three criteria for diagnosing MES, namely: (1) erythrocytosis, (2) a myomatous uterus, and (3) restoration and maintenance of normal hemoglobin levels after hysterectomy. Till 2020, approximately 60 cases of MES are published (1). The diagnosis of our MES case was based on the three diagnostic criteria described above.

On gross inspection, specimens from the patient revealed only small extent of degeneration, which was somewhat an unusual feature considering its large size. As a myoma outgrows its blood supply, it usually undergoes degenerative changes, with hyaline degeneration being the most common manifestation in 60% of the reported cases (4). Therefore, we speculated that the excessive Epo production could have prevented the large broad ligament myoma from undergoing degenerative changes, resulting in its rapid growth.

**Figure 4:**
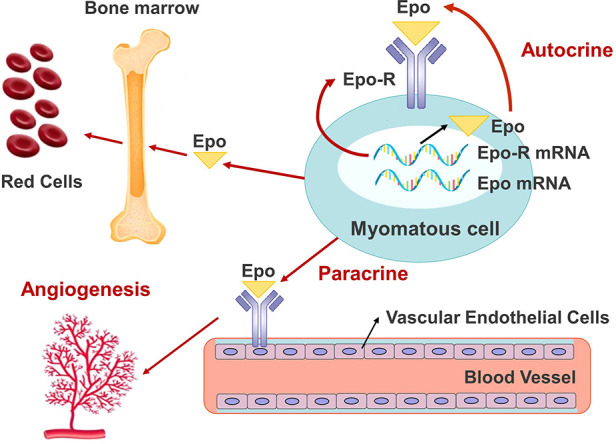
The autocrine, paracrine loop of Epo/Epo-R that resulted in the angiogenesis, excessive growth of the leiomyoma, and erythrocytosis. EPO, erythropoietin; Epo-R, erythropoietin receptor.

In our case, IHC staining revealed diffuse Epo expression throughout the myomatous tissue; however, the endothelial cells were not similarly stained. This finding provides direct evidence that ectopic Epo was produced by the myomatous cells rather than by endothelial cells. In addition, H&E staining demonstrated widespread, highly-matured blood vessels in the myomatous tissue, which was compatible with a vessel maturity score of 5, as defined in previous studies (5). The vessels featured dilated veins and arteries with smooth muscle layers and a luminal space corresponding to ≥50 µm in diameter. Such vascularization is consistent with the known interactions between Epo/Epo-R and the vascular endothelial cells in facilitating neo-angiogenesis (6). These findings explain the process of highly-matured blood vessels formation, and the possible way these vessels protected the leiomyoma from hypoxia and degeneration despite its large size and contributed to the rapid overgrowth (Figure 4).

Epo and Epo-R are present in malignant cells of the cervix, endometrium and ovary. The tumor and vascular endothelial cells are sites responsive to Epo (7). In tumors, the dimeric complex of Epo and Epo-R acts on cells to enhance proliferation and inhibits apoptosis (6). Epo secreted by tumor cells also acts on vascular endothelial cells *via* Epo-R, which activates the mitogenic pathway, EpoR-JAK2-STAT5, and promotes paracrine angiogenesis (6). This loop of autocrine and paracrine between Epo and Epo-R is possibly associated with oncogenesis in the female reproductive organ (8). Studies from the last two decades have confirmed the presence of Epo and Epo-R in myomatous tissues. Asano et al. have reported the presence of Epo mRNA in the myomatous tissues of 95% patients, regardless of erythrocytosis (5). Epo mRNA expressions in myoma specimens are positively correlated with the serum Epo protein level, size of the myomatous uterus, and intra-myomatous vessel maturity in patients with and without MES (5).

In the present case, reduction of the risk of polycythemia-associated thromboembolism was attempted by prophylactic administration of 40 mg enoxaparin prior to the surgery. Unosawa et al. (9) reported a patient with MES who had developed pulmonary embolism. This report supports the importance of recognizing MES and the necessity of prophylactic measures. In previous studies, venous phlebotomy had been performed before the operation in an attempt to normalize the hemoglobin level and reduce the risk of thrombosis. However, Menzies et al. opposed this approach due to the unpredictable blood loss during surgery (10). Therefore, in later studies, blood of the patient was stored after phlebotomy, and autologous blood transfusion was performed as required.

Broad ligament is the most common site for extra-uterine myoma, with an incidence of ≤1% (11). Only two cases of erythrocytosis associated with an extra-uterine leiomyoma have been published in the Pubmed database, including one found at the esophagus and the other presenting as multiple cutaneous leiomyomata (12, 13). To the best of our knowledge, our patient is the first case of MES associated with an intra-ligament leiomyoma. According to the definition, MES involves a “myomatous uterus”, and it turns out that leiomyomata can cause erythropoiesis, regardless of its location. Provided the rarity of MES, and that a broad ligament leiomyoma usually mimics an ovarian tumor under image studies, a correct preoperative diagnosis was challenging in our case.

The incidence of MES is surprisingly low when compared with the high prevalence of uterine leiomyoma. With the report of Epo mRNA present in 95% of leiomyomata (5), it can be inferred that erythrocytosis associated with leiomyoma may actually be present in a larger population than those reported. The under-appreciation of MES can be explained by several factors. First, an elevated RBC count and hemoglobin level can be masked in the presence of menorrhagia; which may partly explain the cause of half MES cases to be diagnosed in menopausal women (1). In addition, the size of the myomatous uterus can be a determining factor. In a review of 57 MES cases, the average size of the myoma was 22.6 ± 10.0 cm in diameter (1). Provided that Epo expression correlates positively to the size of the leiomyoma, it is reasonable to infer that to induce erythrocytosis, a uterine myoma must grow to an extent to generate adequate Epo to produce an abnormally high serum hemoglobin level. In other words, if ectopic Epo expression exists in most leiomyomata, it may only be a matter of degree whether the size of the myoma and the extent of Epo expression can induce erythrocytosis to levels exceeding the upper limit of normal hemoglobin level.

In conclusion, a correct preoperative diagnosis of MES is challenging when it manifests as an extra-uterine mass. As MES usually mimics an ovarian malignancy, a misdiagnosis can lead to unnecessary surgical procedures or even chemotherapy. The risk of polycythemia associated thrombosis also necessitates pre-operative prophylaxis. The coexistence of MES should be considered in the management of all leiomyoma with polycythemia, regardless of its location.

## Data Availability

The original contributions presented in the study are included in the article/Supplementary Material, further inquiries can be directed to the corresponding author/s.
